# Adenosine A2A Receptor Deficiency Up-Regulates Cystatin F Expression in White Matter Lesions Induced by Chronic Cerebral Hypoperfusion

**DOI:** 10.1371/journal.pone.0052566

**Published:** 2012-12-20

**Authors:** Wei Duan, Hong Ran, Zhujuan Zhou, Qifen He, Jian Zheng

**Affiliations:** Department of Neurology, Xinqiao Hospital, Third Military Medical University, Chongqing, China; University of Pecs Medical School, Hungary

## Abstract

In previous studies, we have shown that the inactivation of the adenosine A2A receptor exacerbates chronic cerebral hypoperfusion-induced white matter lesions (WMLs) by enhancing neuroinflammatory responses. However, the molecular mechanism underlying the effect of the adenosine A2A receptor remains unknown. Recent studies have demonstrated that cystatin F, a potent endogenous cysteine protease inhibitor, is selectively expressed in immune cells in association with inflammatory demyelination in central nervous system diseases. To understand the expression of cystatin F and its potential role in the effect of A2A receptor on WMLs induced through chronic cerebral hypoperfusion, we investigated cystatin F expression in the WMLs of A2A receptor gene knockout mice, the littermate wild-type mice and wild-type mice treated daily with the A2A receptor agonist CGS21680 or both CGS21680 and A2A receptor antagonist SCH58261 after chronic cerebral hypoperfusion. The results of quantitative-PCR and western blot analysis revealed that cystatin F mRNA and protein expression were significantly up-regulated in the WMLs after chronic cerebral hypoperfusion. In addition, cystatin F expression in the corpus callosum was significantly increased in A2A receptor gene knockout mice and markedly decreased in mice treated with CGS21680 on both the mRNA and protein levels. Additionally, SCH58261 counteracted the attenuation of cystatin F expression produced by CGS21680 after chronic cerebral hypoperfusion. Moreover, double immunofluorescence staining revealed that cystatin F was co-localized with the activated microglia marker CD11b. In conclusion, the cystatin F expression in the activated microglia is closely associated with the effect of the A2A receptors, which may be related to the neuroinflammatory responses occurring during the pathological process.

## Introduction

Vascular cognitive impairment is defined as any clinical cognitive disorder of cerebrovascular origin [1]. Chronic reduction in cerebral blood flow is one of the most common causes of vascular cognitive impairment in the elderly. This reduction typically manifests pathologically with the development of ischemic white matter lesions (WMLs). Strong evidence indicates that a neuroinflammatory mechanism is correlated with the progression of ischemic WMLs induced through chronic cerebral hypoperfusion [2], [3]. In addition, activated microglia and increased proinflammatory cytokine production play key roles in neuroinflammation in white matter after chronic cerebral hypoperfusion in the brain [4].

As resident monocytic-macrophage cells within the brain, microglia often act as cytotoxic effector cells that release proinflammatory cytokines and other harmful substances, such as proteases, reactive oxygen intermediates and nitric oxide [5], [6]. Recent studies have shown that cystatin F (CF), a potent endogenous cysteine protease inhibitor, is primarily expressed in activated microglia in central nervous system diseases but is not expressed in the normal brain [7]. More importantly, CF expression was dramatically up-regulated in regions of white matter injury that occur in a variety of demyelinating diseases of the central nervous system [7], [8]. CF is a member of the papain-like C1 family of cysteine proteases, which also includes the endosomal/lysosomal cathepsins [9], and is selectively expressed in immune cells and tissues [10], [11]. In addition, an increasing number of studies have revealed that CF exhibits a proinflammatory role through the increased production of active proinflammatory cytokines in inflammatory responses [12] and regulation of the differentiation and maturation of immune cells [10]. Accordingly, up-regulated CF expression in activated microglia might induce the neuroinflammation in WMLs associated with the exacerbation demyelinating diseases of the central nervous system.

As an endogenous neuromodulator in the brain, adenosine binds to four specific adenosine receptor subtypes, A1, A2A, A2B and A3, and exerts immunomodulating and protective effects in a wide variety of the neuroinflammatory responses after brain injury. Recently, the activation of the A2A receptor has been shown to protect against inflammatory damage through the inhibition of proinflammatory cytokine production in immune and inflammatory cells in animal models of several neuroinflammatory diseases [13], [14], [15]. Moreover, in a previous study, we have shown that A2A receptor inactivation using a global gene knockout aggravated the WMLs induced through chronic cerebral hypoperfusion, and the exacerbation of WMLs was associated with increased glial activation and elevated proinflammatory cytokine production [16]. However, the signal pathways involved in the effect of the adenosine A2A receptor on chronic cerebral hypoperfusion-induced WMLs have not been elucidated. It has been demonstrated that the activation of the adenosine A2A receptor inhibits the synthesis and release of lysosomal cathepsins through the up-regulation of cyclic AMP (cAMP)-regulated element binding protein (CREB) [17], [18]. In addition, some experiments have revealed that the activity of CF is activated by the proteolytic cleavage of cathepsins in the endosomal/lysosomal system before engaging the target protease [12], [19]. Thus, we hypothesize that CF plays a role in the adenosine A2A receptor-mediated exacerbation of ischemic WMLs induced through chronic cerebral hypoperfusion.

In this study, we examined CF gene and protein expression in WMLs induced through chronic cerebral hypoperfusion. Moreover, we evaluated the gene and protein expression levels of CF in the corpus callusom of adenosine A2A receptor gene knockout (KO) and wild-type (WT) mice and WT mice treated with the A2A receptor agonist CGS21680 or both CGS21680 and A2A receptor antagonist SCH58261. Our results demonstrated that the mRNA and protein levels of CF significantly increased in the WMLs that were induced by chronic cerebral hypoperfusion. Moreover, knocking out the adenosine A2A receptor gene up-regulated the expression of cystatin F in the WMLs after chronic cerebral hypoperfusion, whereas A2A receptor activation down-regulated it.

## Materials and Methods

### Ethics Statement

The animals were housed and maintained at room temperature (22±2°C) with free access to food and water. All mice care and experimentation were approved by the Institutional Animal Care and Use Committee of the Third Military Medical University (SYXK-PLA-2007035). Efforts were made to minimize animal suffering and reduce the number of animals used. All surgery was performed under sodium pentobarbital anesthesia. All mice were killed by cervical dislocation.

### Animals

The Boston University School of Medicine (Boston, USA) generously provided C57BL/6 heterozygote A2A receptor gene KO mice. Ten-week-old male C57BL/6 A2A receptor KO mice and their WT littermates (weighing 24–29 g) were used for this study. Genomic DNA was isolated from the tails, and the genotype of each mouse was determined using PCR analysis as previously described [20], [21].

### Bilateral Common Carotid Artery Stenosis Surgery

Bilateral common carotid artery stenosis (BCAS) surgery was performed according to the method of Shibata et al. [22]. Briefly, microcoils were obtained from (Invitrotech CO, Osaka, Japan). The microcoils were composed of piano wire with an inner diameter of 0.18 mm. The mice were anesthetized with an intraperitoneal injection (i.p.) of sodium pentobarbital (50 mg/kg). Using a midline cervical incision, both common carotid arteries (CCAs) were exposed and freed from their sheaths. Two 4-0 silk sutures were placed around the distal and proximal regions of the right CCA. Subsequently, the artery was gently lifted and placed between the loops of the microcoil directly below the carotid bifurcation. The microcoil was twined around the CCA. After 30 min, another microcoil of the same size was twined around the left CCA. Rectal temperature was maintained between 36.5 and 37.5°C.

### Experimental Groups

In the experiments in which SCH58261 or CGS21680 were systemically injected, the mice were randomly allocated into the following four groups. (1) *WT group*: wild-type mice were subjected to BCAS surgery. (2) *KO group*: adenosine A2A receptor gene knockout mice were subjected to BCAS surgery. (3) *WC group*: wild-type mice were subjected to the BCAS surgery, then given CGS21680 (Tocris Bioscience, U.K.) at the dose of 0.25 mg/kg (i.p.) 30 min after surgery and then repeatedly treated every 24 hours for 2, 4 and 6 weeks. (4) *CGS+SCH group*: wild-type mice were subjected to BCAS surgery, then given SCH58261 (Tocris Bioscience, U.K.) at a dose of 0.1 mg/kg (i.p.) 10 min following surgery and CGS21680 (Tocris Bioscience, U.K.) at a dose of 0.25 mg/kg (i.p.) 30 min after surgery [23], then repeatedly treated at 20-min interval every 24 hours for 2, 4 and 6 weeks. The dose of SCH58261 used in the present study was selected based on preliminary experiments that tested a wider range of SCH58261 doses on a number of animals. Several studies have indicated that a SCH58261 dose of 0.1 mg/kg does not have a peripheral effect on heart rate or systemic blood pressure but does exert a neuroprotective effect against a variety of brain insults [24], [25], [26].

### Cerebral Blood Flow Measurement

Under deep anesthesia with sodium pentobarbital (50 mg/kg, i.p.), the skin overlying the right skull was reflected. Using dental resin, a plastic guide cannula (3-mm outer diameter, 2-mm inner diameter, and 4-mm length) was fixed perpendicularly to the skull at 1 mm posterior and 2.5 mm lateral to the bregma to serve as a laser-Doppler flowmetry probe. A 2.0 mm straight probe (OmegaFLO-N1, Neuroscience Inc, Wisconsin, U.S.) was placed through the guide cannula to record cerebral blood flow (CBF). The baseline CBF recordings were obtained prior to and immediately after surgery. The CBF values were expressed as a percentage of the baseline value.

### Histochemical Evaluation of WMLs

At 2, 4, and 6 weeks after BCAS, the mice were deeply anesthetized with pentobarbital and transcardially perfused with 0.9% saline, followed by 4% paraformaldehyde in 0.1 M phosphate buffer (PB, pH 7.4). The brains were harvested, postfixed for 12 h in 4% paraformaldehyde in 0.1 M PB (pH 7.4), and subsequently stored in 30% sucrose in 0.1 M PB (pH 7.4). Serial coronal sections (10 or 30 µm), spanning from the anterior region of the corpus callosum (bregma 0.26 mm) to the anterior region of the hippocampus (bregma 0.94 mm) according to the mouse brain atlas [27], were cut on a cryostat. Every fifth section (180 µm) was cut at 10 µm and processed for Klüver-Barrera (KB) staining. Two independent investigators blinded to the treatment type assessed the severity of the WMLs as normal (grade 0), disarrangement of the nerve fibers (grade 1), formation of marked vacuoles (grade 2), or disappearance of myelinated fibers (grade 3) [22]. The WMLs were evaluated in three regions: the corpus callosum, internal capsule, and optic tract.

### Determination of Cystatin F mRNA Level by Quantitative-PCR

The mRNA levels of cystatin F of each group were evaluated as previously described [28], [29]. At 2, 4, and 6 weeks after surgery (n = 6 at each time point), the animals were anesthetized with 1% sodium pentobarbital (50 mg/kg, i.p.); the brain was quickly harvested, and the entire corpus callosum was carefully dissected. The tissues were snap-frozen in liquid nitrogen and stored at −80°C. Half of the corpus callosum in each animal was used for quantitative PCR analysis, and the other half was used for Western blot analysis. For the quantitative PCR analysis, the frozen tissues were homogenized in Trizol reagent (Invitrogen, Carlsbad, CA, USA). Total RNA was extracted from the Trizol homogenates according to the manufacturer's guidelines. The RNA concentration and quality were evaluated spectrophotometrically. The total RNA was used to reverse transcribe cDNA. The quantitative PCR amplification of the cDNA was performed in triplicate using a SYBR Green kit (TaKaRa Bio Inc., Dalian, China). Glyceraldehyde phosphate dehydrogenase (GAPDH) served as the endogenous control gene. The primers for CF and GAPDH are shown in Table 1.

**Table 1:** Primer characteristics.

	Sense Primer (5′-3′)	Antisense Primer (3′-5′)
CF	ACCAATAACCCAGGAGTGCTTA	TGACCCAGACTTCAGAGTAGCA
GAPDH	ACCCATCACCATCTTCCAGGAG	GAAGGGGCGGAGATGATGAC

### Examination of Cystatin F Protein Expression Using Western Blot Analysis

The frozen tissues were transferred to ice-cold buffer containing 0.1 mol/L NaCl, 0.05 mol/L Tris–HCl (pH 7.6), 0.001 mol/L EDTA (pH 8.0), 0.1% Tween-20, aprotinin (1 µg/mL), and PMSF (100 µg/mL) and homogenized on ice. The homogenate was centrifuged at 10,000 g for 10 min, and the supernatant was stored at 4°C. Total protein concentrations were determined with a UV spectrophotometer using a modified Bradford assay (Beckman Coulter, Fullerton, CA, USA). Equal amounts of protein from each sample (100 µg) were mixed with 30–35 µL of sample buffer and boiled for 5 min. The samples were separated using electrophoresis on 12% polyacrylamide gels. The separated proteins were transferred onto a polyvinylidene fluoride (PVDF) membrane at 60 mA for 2.0 h. After blocking with 3% BSA in Tris-buffered saline containing 0.1% Tween 20 (TBS-T), pH 7.6, the membranes were incubated overnight at 4°C with a goat polyclonal anti-CF antibody (1∶200, Santa Cruz Biotechnology, Inc, USA). The next day, the membranes were washed with TBST and incubated for 1 h at room temperature with horseradish peroxidase-conjugated anti-goat antibodies (1∶2000, Zhongshan Biotechnology, Beijing, China). An endogenous control protein, β-actin, was included in each Western blot experiment. The PVDF membrane was fixed and visualized. The optical densities of specific bands were scanned and measured using image analysis software (Quantity One 4.4.0.36, USA) and normalized to β-actin.

### Immunofluorescence Staining of Cystatin F

For immunofluorescence staining, coronal sections (30 µm) were treated with 3% H2O2 in 0.01 M phosphate-buffered saline (PBS) and preincubated in 5% normal goat serum. The samples were subsequently incubated in a primary antibody solution containing goat anti-CF (1∶50, Santa cruz Biotechnology, Inc, USA) and rat anti-CD11b (1∶100, Chemicon, Colorado, U.S.) antibodies for 1 h at 37°C, followed by further incubation at 4°C overnight. After washing, the samples were incubated in a secondary antibody solution containing DyLight 649-conjugated rabbit anti-goat or FITC-conjugated donkey anti-rat biotinylated IgG (1∶100, Zhongshan Biotechnology, Beijing, China) for 1 h at 37°C. Finally, 4′,6-diamidino-2-phenylindole (DAPI, 10 ug/ml, Beyotime, China) was used to counterstain the cell nuclei. The fluorescent sections were observed and photographed using a confocal laser-scanning microscope (TCSTIV; Leica, Nussloch, Germany).

To evaluate the immunofluorescence staining, a semi-quantitative analysis was performed as previously reported [30]. A Leica TCSTIV microscope was used to examine each slice, and high-power non-overlapping fields (0.5 mm×0.5 mm width, 0.25 mm^2^/field of view) were defined in the WMLs, which include three regions: the corpus callosum, the internal capsule, and the optic tract. The labeled cells were counted per visual field and expressed as the number of cells (± S.E.M.) per 0.25 mm^2^ area. Only the cells whose nuclei were counterstained with DAPI and that were clearly positive for the antigen of interest were identified as immunopositive cells and included in the analysis.

### Data Analysis and Statistics

The data are expressed as the means ± S.E.M. Statistical analyses were performed using SPSS for Windows (SPSS 15.0, SPSS Inc., Chicago, IL, USA). For evaluation of the CBF before and after surgery, significance of differences between groups were analyzing using repeated measures analysis of variance (ANOVA) followed by a post-hoc test for multiple comparisons. For the quantitative-PCR, western blot and immunofluorescence staining experiments, the data were analyzed using two-way ANOVA followed by the LSD test for multiple comparisons, and *p*<0.05 was considered significant.

## Results

### Evaluation of CBF after Surgery

The CBF values were recorded for all animals before and after surgery using laser-Doppler flowmetry. The mean CBF value was significantly decreased by 62.8±11.9% in all groups. No marked differences in CBF were observed between groups.

### Histochemical Evaluation of White Matter

**Figure 1:**
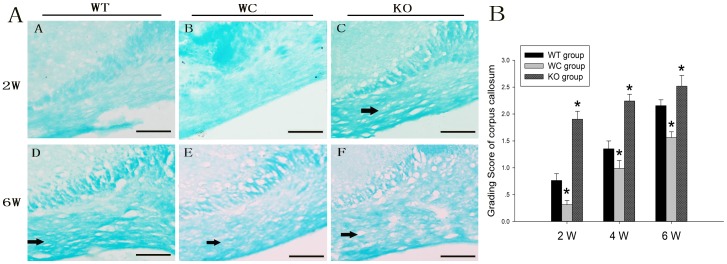
Histologic evaluation of WMLs in the WT, WC and KO groups. (A) KB staining in the corpus callosum at 2 and 6 weeks after BCAS. The disarrangement of the nerve fibers and formation of marked vacuoles were observed after BCAS (arrows). (B) Temporal profiles of the grading scores in WMLs in the corpus callosum at 2, 4 and 6 weeks after BCAS. (Scale bar: 100 µm; n = 6, * *p*<0.05 compared with WT group at the same time point).

White matter rarefaction was detected at 2, 4 and 6 weeks in all groups after BCAS. The most significant changes were observed in the medial region of the corpus callosum adjoining the lateral ventricle (Fig. 1A). Moderate changes were observed in the internal capsule, and less severe changes were observed in the optic tract (data not shown). Fig. 1B shows the grading score of WMLs in the corpus callosum of each group. Compared with the WT group, the WMLs in the corpus callosum were significantly more severe in the KO group at 2, 4 and 6 weeks after BCAS (*p*<0.05). In contrast, the white matter rarefaction grading score in the WC group was significantly decreased compared with the WT group at 2, 4 and 6 weeks after BCAS (*p*<0.05). Moreover, the white matter rarefaction in each group was significantly more severe at 4 and 6 weeks than 2 weeks (*p*<0.05).

### CF mRNA Levels in the Corpus Callosum

**Figure 2:**
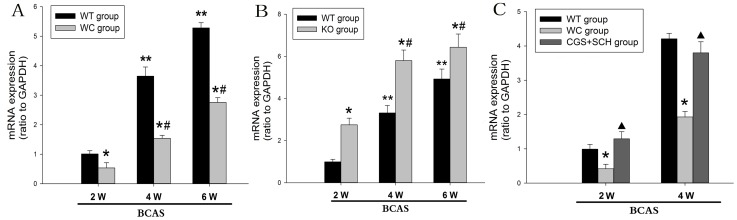
Evaluation of CF mRNA expression in the corpus callosum of the WT, WC, KO and CGS+SCH groups at each time pointafter BCAS. (A) Comparison of CF mRNA levels in the corpus callosum between the WT and WC groups. (B) Comparison of the CF mRNA levels in the corpus callosum between the WT and KOgroups. (C) Comparison of the CF mRNA levels in the corpus callosum among WT, WC and CGS+SCH groups. [n = 6; * *p*<0.05 compared with the WT group at the same time point; # *p*<0.05 compared with the same group (WC or KO group) at 2 weeks; ** *p*<0.05 compared with the WT group at 2 weeks;▴ *p*<0.05 compared with WC group at the same time point].

The CF mRNA levels in the corpus callosum of each group were examined using quantitative-PCR at 2, 4 and 6 weeks after BCAS. Fig. 2A shows the CF mRNA levels in the corpus callosum of the WT and WC groups. The CF mRNA levels in the WT group at 4 and 6 weeks were significantly increased compared with the same group at 2 weeks (*p*<0.05). Similarly, the CF mRNA levels in the WC group at 4 and 6 weeks were significantly increased compared with the same group at 2 weeks (*p*<0.05). Moreover, the CF mRNA levels in the WC group were significantly decreased compared with the WT group at 2, 4 and 6 weeks (*p*<0.05). As shown in Fig. 2B, the CF mRNA levels in the KO group at 4 and 6 weeks were significantly increased compared with the same group at 2 weeks (*p*<0.05). In addition, the CF mRNA levels in the KO group were significantly higher than the WT group at 2, 4 and 6 weeks (*p*<0.05). Fig. 2C shows the effect of the adenosine A2A receptor antagonist SCH58261 plus the agonist CGS21680 on CF expression. There were no significant differences in the CF mRNA levels between the WT group and the CGS+SCH group treated with CGS21680 and SCH58261. Additionally, the CF mRNA levels in the CGS+SCH group were significantly increased compared with those in the WC group at 2 and 4 weeks after BCAS.

### Western Blot Analysis of CF Protein Expression in the Corpus Callosum

**Figure 3:**
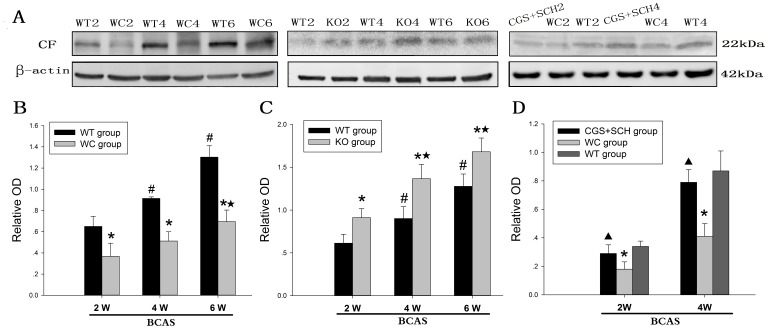
Western blot analysis of CF protein levels in corpus callosum in the WT, WC, KO and CGS+SCH groups at each time point after BCAS. (A) Representative immunoblot bands; (B–D) densitometric analysis of total homogenates between the WT and WC groups (B); the WT and KO groups (C); the WT, WC and CGS+SCH group (D). Molecular weight band detected using anti-CF antibodies is shown at the expected size of 22 kDa. Relative OD is expressed as the mean ± SEM. [n = 6; * *p*<0.05 compared with the WT group at the same time point; # *p*<0.05 compared with the WT group at 2 weeks; ★ *p*<0.05 compared with the same group (WC or KO group) at 2 weeks; ▴ *p*<0.05 compared with WC group at the same time point].

We examined the level of CF expression in the corpus callosum using western blot analysis. As shown in Fig. 3A, CF was present as a 22-kDa band. Consistent with the data obtained from our PCR analysis, the results of the semi-quantitative densitometric analysis indicated that the CF protein levels in the WT and KO groups at 4 and 6 weeks were significantly higher than those at 2 weeks (*p*<0.05). Notably, a marked increase in the CF protein level in the WC group was only observed at 6 weeks compared with the same group at 2 weeks (*p*<0.05). The CF protein level was significantly decreased in the WC group at 2, 4 and 6 weeks compared with the WT group (*p*<0.05). In contrast, the CF protein level was significantly higher in KO group than the WT group at 2, 4 and 6 weeks (*p*<0.05). Furthermore, we also found that there were no significant differences in the CF protein levels between the WT group and the CGS+SCH group treated with CGS21680 and SCH58261. Additionally, the CF protein levels in the CGS+SCH group were significantly increased compared with those in the WC group at 2 and 4 weeks after BCAS (Fig. 3D).

### Cystatin F Immunoreactivity in WMLs

**Figure 4:**
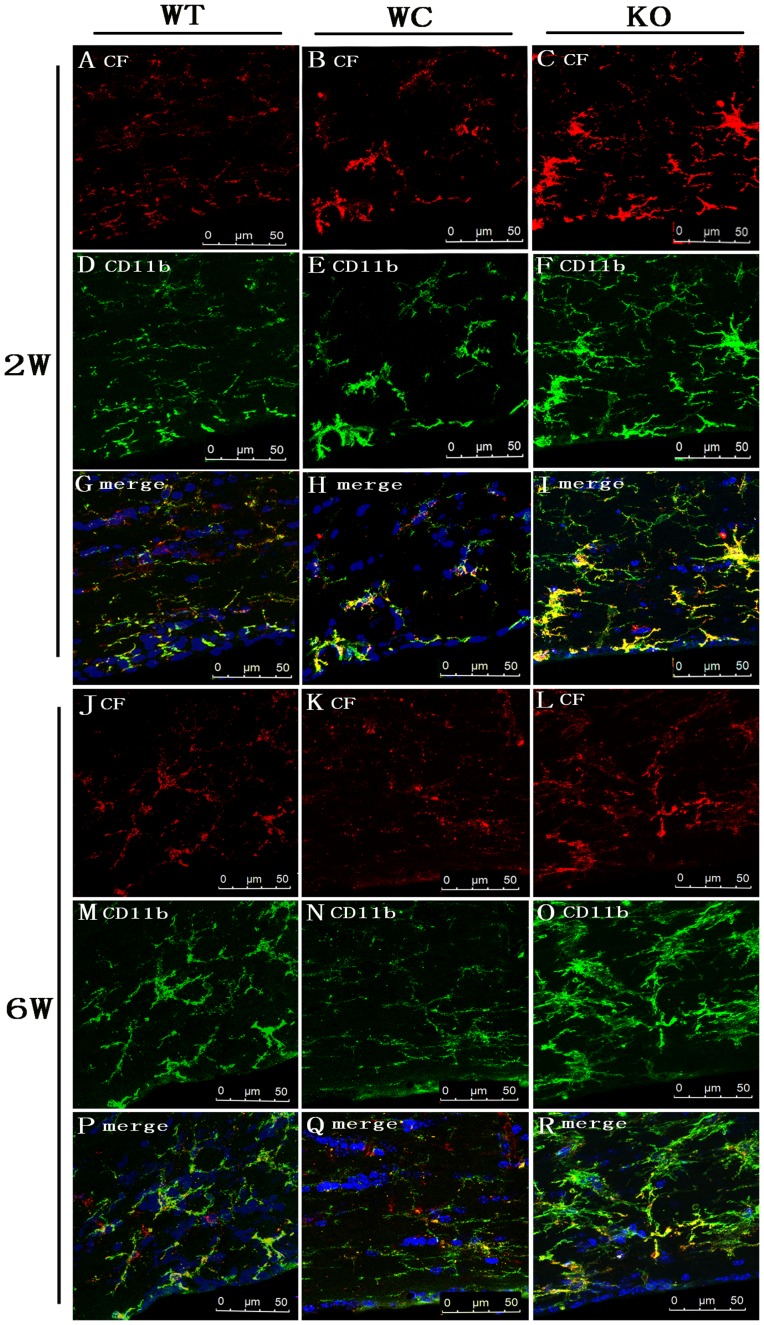
CF immunoreactivity in the corpus callosum of the WT, WC and KO groups at 2 and 6 weeks after BCAS. Representative confocal images showing abundant CD11b positive cells (green) were observed in the corpus callosum in all groups after BCAS (D–F, M–O). The expression of CF (red) was also observed in the corpus callosum in all groups after BCAS (A–C, J–L). DAPI (blue) was used to counterstain the cell nuclei. The merged images show the co-localization of CF (red) with activated microglia (green) in the corpus callosum in all groups after BCAS (G–I, P–R). (Scale bar = 50 µm; n = 6).

**Figure 5:**
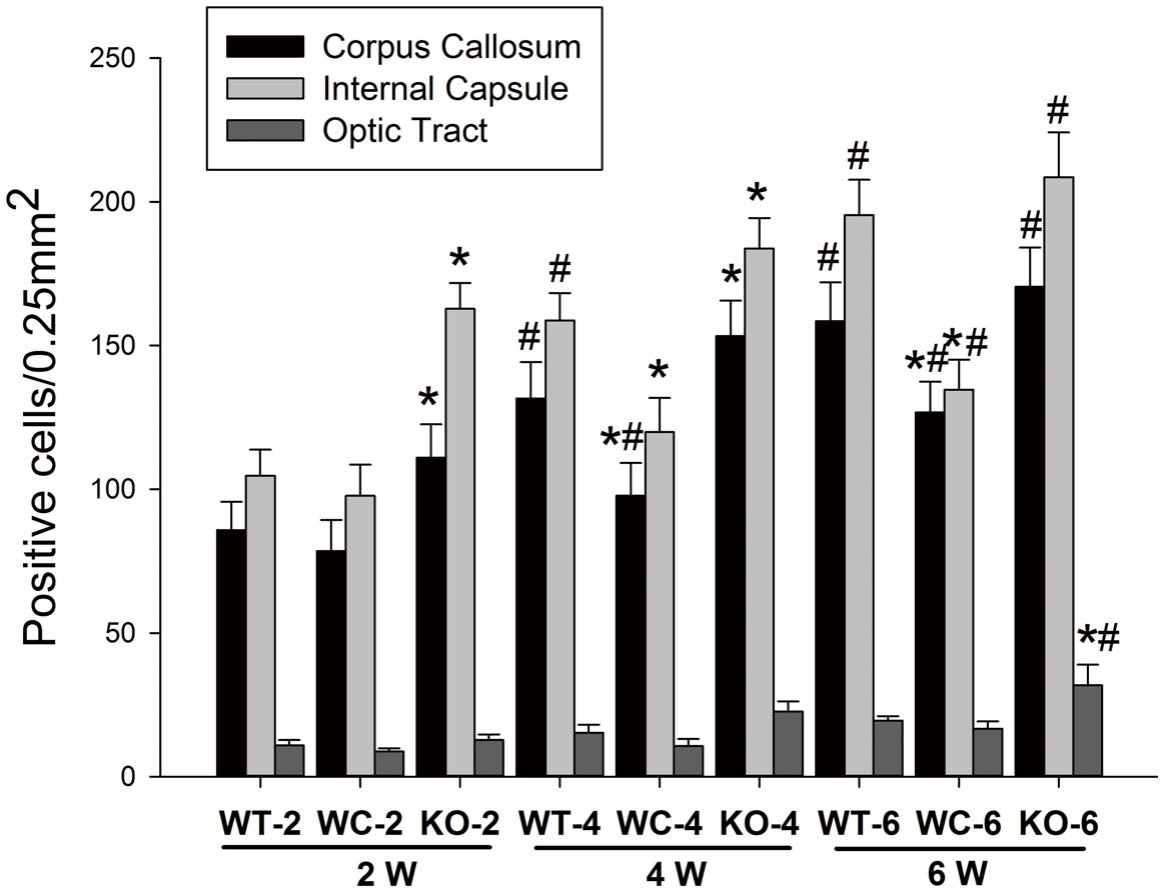
Temporal profiles of the density of double immunopositive cells for CF and CD11b in the corpus callosum, internal capsule and optic tract from the WT, WC and KO groups at 2, 4 and 6 weeks after BCAS. The number of double immunopositive cells per 0.25-mm^2^ in WM lesions of each group are expressed as the mean ± SEM. [n = 6; * *p*<0.05 compared with the WT group at the same time point; # *p*<0.05 compared with the same group (WT, KO or WC group) at 2 weeks].

After BCAS, the CF expression was detected in WMLs at 2, 4 and 6 weeks using immunofluorescence staining. The highest expression of CF was observed in the corpus callosum and internal capsule after BCAS. Fig. 4 shows the immunoreactivity of CF in the corpus callosum of each group at 2 and 6 weeks. Double-labeling staining revealed the co-localization of CF with the microglia marker CD11b in WMLs. As shown in Fig. 5, numerical densities of CF immunopositive cells in the corpus callosum and internal capsule were significantly more increased at 4 and 6 weeks than 2 weeks (*p*<0.05). However, a significant increase in the numerical densities of CF immunopositive cells in the optic tract was only observed at 6 weeks (*p*<0.05). Compared with the WT group, the numerical densities of CF immunopositive cells in the corpus callosum and internal capsule of the KO group were markedly increased at 4 and 6 weeks after BCAS (*p*<0.05). However, the numerical densities of CF immunopositive cells in the corpus callosum of the WC group were significantly lower than the WT group at 2, 4 and 6 weeks after BCAS (*p*<0.05).

## Discussion

In the present study, we showed that high levels of CF mRNA and protein were expressed in the WMLs after chronic cerebral hypoperfusion. Compared with 2 weeks after chronic cerebral hypoperfusion, the expression of cystatin F in the WMLs was significantly more increased at both the mRNA and protein levels at 4 and 6 weeks when the occurrence of WMLs were significantly more severe. We further showed that a deficiency in the adenosine A2A receptor up-regulated CF mRNA and protein expression, whereas the administration of the adenosine A2A receptor agonist CGS21680 down-regulated CF mRNA and protein expression in the WMLs after chronic cerebral hypoperfusion. Moreover, the adenosine A2A receptor antagonist SCH58261 counteracted the down-regulation of cystatin F expression that was induced by CGS21680 in the WMLs after chronic cerebral hypoperfusion. Furthermore, double-labeling staining for CF and the microglia marker CD11b indicated that CF was expressed in the activated microglia of the WMLs induced through chronic cerebral hypoperfusion.

Recently, accumulating evidence has shown that CF expression is observed in a variety of tissues, and particularly high in the cells and tissues of the immune system, such as the thymus and spleen, monocytes, dendritic cells, T-cells and NK cells, etc [19], [31]. However, little is known about the expression of CF in the central nervous system. Increasing evidence has demonstrated that CF is expressed in activated microglia in demyelinated WMLs mouse models, such as cuprizone-demyelination models, myelin oligodendrocyte glycoprotein (MOG)-induced experimental autoimmune encephalomyelitis (EAE) demyelination models and the hemizygous proteolipid protein 1 (Plp1) transgenic mouse; notably, CF expression is completely absent in the normal brain [7]. Moreover, CF has also been observed in the spinal cord tissues of multiple sclerosis and Aicardi-Goutières patients [8]. It is well known that the microglia in the brain belong to the monocytic-macrophage lineage and serve as brain immune cells that evoke neuroinflammatory responses [32], [33]. Thus, characterizing the expression and cellular location of CF in WMLs induced through chronic cerebral hypoperfusion will provide further insight into a putative role for this protein in the development of myelin sheath injury. In the present study, the mRNA and protein levels of CF in the corpus callosum were dramatically increased at 2, 4 and 6 weeks after chronic cerebral hypoperfusion and markedly increased with the development of demyelination and the activation of microglia. Moreover, double immunofluorescence staining revealed that CF co-localized with the activated microglia marker CD11b in WMLs induced through chronic cerebral hypoperfusion. However, CF was not detected in normal brain tissues (data not shown). In addition, we counted the number of CF immunopositive cells in the WMLs and observed that the total number of CF immunopositive cells was significantly increased in the corpus callosum and internal capsule after chronic cerebral hypoperfusion. Thus, these results suggested that CF expression is up-regulated in activated microglia in WMLs induced through chronic cerebral hypoperfusion. More importantly, up-regulated CF might be associated with the exacerbation of WMLs after chronic cerebral hypoperfusion.

It has been widely accepted that adenosine A2A receptors mediate potent anti-inflammatory effects in many tissues of the central nervous system [34], [35]. The results from previous studies in our laboratory have demonstrated that the genetic deletion of the adenosine A2A exacerbates the WMLs induced by chronic cerebral hypoperfusion [16]. Accordingly, we tested the hypothesis that up-regulated CF might participate in the effect of adenosine A2A receptor on chronic cerebral hypoperfusion-induced WMLs. In the present study, using adenosine A2A receptor gene knockout mice and the A2A receptor agonist CGS21680, we demonstrated that the mRNA and protein expression of cystatin F in the corpus callosum was significantly enhanced in A2A receptor knockout mice at 2, 4 and 6 weeks after chronic cerebral hypoperfusion. In contrast, the CF mRNA and protein expression in corpus callosum was significantly decreased in mice treated with the A2A receptor agonist CGS21680 at 2, 4 and 6 weeks after chronic cerebral hypoperfusion. Our study also revealed that the CF mRNA and protein levels in the corpus callosum in the CGS+SCH group were strikingly increased compared with those in the WC group, which indicated that the adenosine A2A receptor antagonist SCH58261 counteracted the down-regulation of cystatin F expression produced by the A2A receptor agonist CGS21680 after chronic cerebral hypoperfusion and confirmed that the effect of CGS21680 on CF expression acted through activation of the adenosine A2A receptor. Moreover, the results of immunofluoresence analysis showed that the A2A receptor gene knockout markedly increased the number of CF immunopositive cells in WMLs induced through chronic cerebral hypoperfusion, whereas the administration of the A2A receptor agonist CGS21680 significantly decreased the number of CF immunopositive cell in WMLs induced through chronic cerebral hypoperfusion. Taken together, these findings demonstrated that adenosine A2A receptor deficiency up-regulated CF mRNA expression and protein synthesis in WMLs induced by chronic cerebral hypoperfusion, whereas the adenosine A2A receptor agonist CGS21680 down-regulated CF expression in WMLs after chronic cerebral hypoperfusion.

The molecular mechanism underlying CF expression regulated by adenosine A2A receptor in ischemic WMLs need to be elucidated. It has been well established that the activation of the adenosine A2A receptor inhibits the synthesis and release of lysosomal cathepsins through the Gs protein-cyclic AMP (cAMP)-regulated element binding protein (CREB) signaling pathway [17]. It is also widely accepted that cysteine cathepsins are the primary proteases involved in a variety of pathological processes [36], [37]. CF has been identified as a major endogenous lysosomal cysteine protease inhibitor, and its potential target is cathepsins [38], [39]. The results of recent studies have suggesed that a proteolytic cleavage event is required to convert inactive CF into an active cathepsin inhibitor [12], [19]. Accordingly, we speculate that an adenosine A2A receptor–CREB–cathepsin signaling pathway might participate in the up-regulation of CF expression in the WMLs of adenosine A2A knockout mice after chronic cerebral hypoperfusion.

Until recently, the potential roles of CF in the adenosine A2A receptor activation-mediated exacerbation of WMLs after chronic cerebral hypoperfusion were unclear. Numerous studies have supported a role for CF in immune responses and the regulation of immune cell activity. For example, CF mRNA levels are significantly upregulated during the maturation and activation of immune cells from quiescent precursors [19], [40]. CF modulates the specific immune response through the inhibition of lysosomal cathepsin, which plays a role in immune cell activation, adhesion and transmigration [39], [41]. CF enhances the production of active pro-inflammatory molecules, increases the expression of inducible nitric oxide synthase and up-regulates nitric oxide production [42]. Therefore, CF has been implicated as an important proinflammatory molecule in inflammatory responses. Future studies will be needed to elucidate the role of CF in the anti-inflammatory effect of the A2A receptor on the WMLs produced by chronic cerebral hypoperfusion.

In conclusion, the results of this study demonstrated that CF mRNA and protein expression was up-regulated in WMLs induced by chronic cerebral hypoperfusion. More importantly, the adenosine A2A receptor deficiency increased the transcription and expression of CF in the corpus callosum after chronic cerebral hypoperfusion, whereas the activation of A2A receptors reduced CF expression under these same conditions. Additionly, CF was expressed in activated microglia in WMLs induced by chronic cerebral hypoperfusion. These results provide further insight into the molecular anti-inflammatory mechanisms of A2A receptor activation in WMLs after chronic cerebral hypoperfusion. Further studies should be conducted to identify the signal pathway that mediates the effects of the adenosine A2A receptor-mediated activation of CF expression.
